# Sex-specific effects of a parasite on stress-induced freezing behavior in a natural beetle-nematode system

**DOI:** 10.1371/journal.pone.0281149

**Published:** 2023-03-14

**Authors:** Andrew K. Davis, Richard R. E. Ladd, Farran Smith, Anna Shattuck

**Affiliations:** 1 Odum School of Ecology, University of Georgia, Athens, GA, United States of America; 2 Biological Sciences, University of Georgia, Athens, GA, United States of America; 3 Tulane University, New Orleans, LA, United States of America; University of Pretoria, SOUTH AFRICA

## Abstract

Some animals react to predation threats or other stressors by adopting a freezing posture in an attempt to avoid detection, and the duration of this behavior usually corresponds with individual personality, such that timid individuals freeze longer. Despite decades of research on this or related behaviors (thanatosis), never has the impact of parasitism been considered. Parasites could prolong the duration, if hosts are less motivated to move (i.e. lethargic), or they could reduce it, if hosts are motivated to forage more to compensate for energy drain. We examined this behavior within a natural beetle-nematode system, where hosts (horned passalus beetles, *Odontotaenius disjunctus*) are parasitized by a nematode, *Chondronema passali*. We exposed beetles (n = 238) to four stressors in our lab, including noise, vibration, light and inversion, and recorded how long they adopt a frozen stance. Afterward, we determined nematode burdens, which can range from dozens to hundreds of worms. Beetles tended to freeze for 20 seconds on average, with some variation between stressors. We detected no effect of beetle mass on the duration of freezing, and this behavior did not differ in beetles collected during the breeding or non-breeding season. There was a surprising sex-based difference in the impact of nematodes; unparasitized females remained frozen twice as long as unparasitized males, but for beetles with heavy nematode burdens, the opposite was true. From this we infer that heavily parasitized females are more bold, while males with heavy burdens would be more timid. The explanation for this finding remains elusive, though we can rule out many possibilities based on prior work on this host-parasite system.

## Introduction

Across the animal kingdom, different species have evolved a wide variety of anti-predator strategies that fit their lifestyle, body design and behavior. When directly faced with a predator or perceived threat, some animals react by feigning death as an attempt to fool the predator [1–4]. In some scientific disciplines this is referred to as thanatosis [5,6] or tonic immobility [7,8]. There can be many forms of this behavior, such as curling the abdomen [i.e. pillbugs, 9], playing dead [i.e., snakes, 10] or stiffening the appendages [crickets, 11]. An alternative strategy is to attempt to avoid detection altogether, and simply freeze, or remain motionless, so the predator cannot detect any vibration or sound of the prey. One paper describes this as simply “quiescence” [12]. In a recent study of beetle behavior, researchers concluded that individuals of the same species can even display different strategies (freeze or feign death) for different threats [13]. Collectively, these anti-predator behaviors have been well-studied, especially within the entomological literature, with much research devoted to understanding the range of triggers that induce the behavior(s) [14], the factors that influence their extent [6,15] and even the successfulness of it in actually promoting survival [1]. Distinctly lacking from this body of work are investigations into how *parasites* might influence this behavior. In fact, in a thorough review of the topic of thanatosis (of 91 studies), no mention was made of parasites [8].

Parasites, by definition, utilize their hosts resources to grow and/or reproduce, and in so doing usurp the host energy stores [16]. This energy drain could motivate hosts to increase foraging activity [17]. Indeed, starvation trials across multiple species have consistently shown that hunger is a strong motivator and reduces the duration and/or and likelihood that animals will even initiate freezing in response to a threat [18–20]. In other words, hungrier animals are more motivated to resume activity and/or foraging, despite the risk of predation. Alternatively, given that infections can often result in lethargy and loss of mobility [21], infected hosts could actually be more likely to remain in place longer when exposed to a threat. So, given the sheer ubiquity of parasites in the animal kingdom [22], and this obvious potential for them to influence host anti-predator behavior (positively or negatively), it is a wonder that this issue has not yet been addressed, despite decades of research on this topic [23–26].

**Fig 1:**
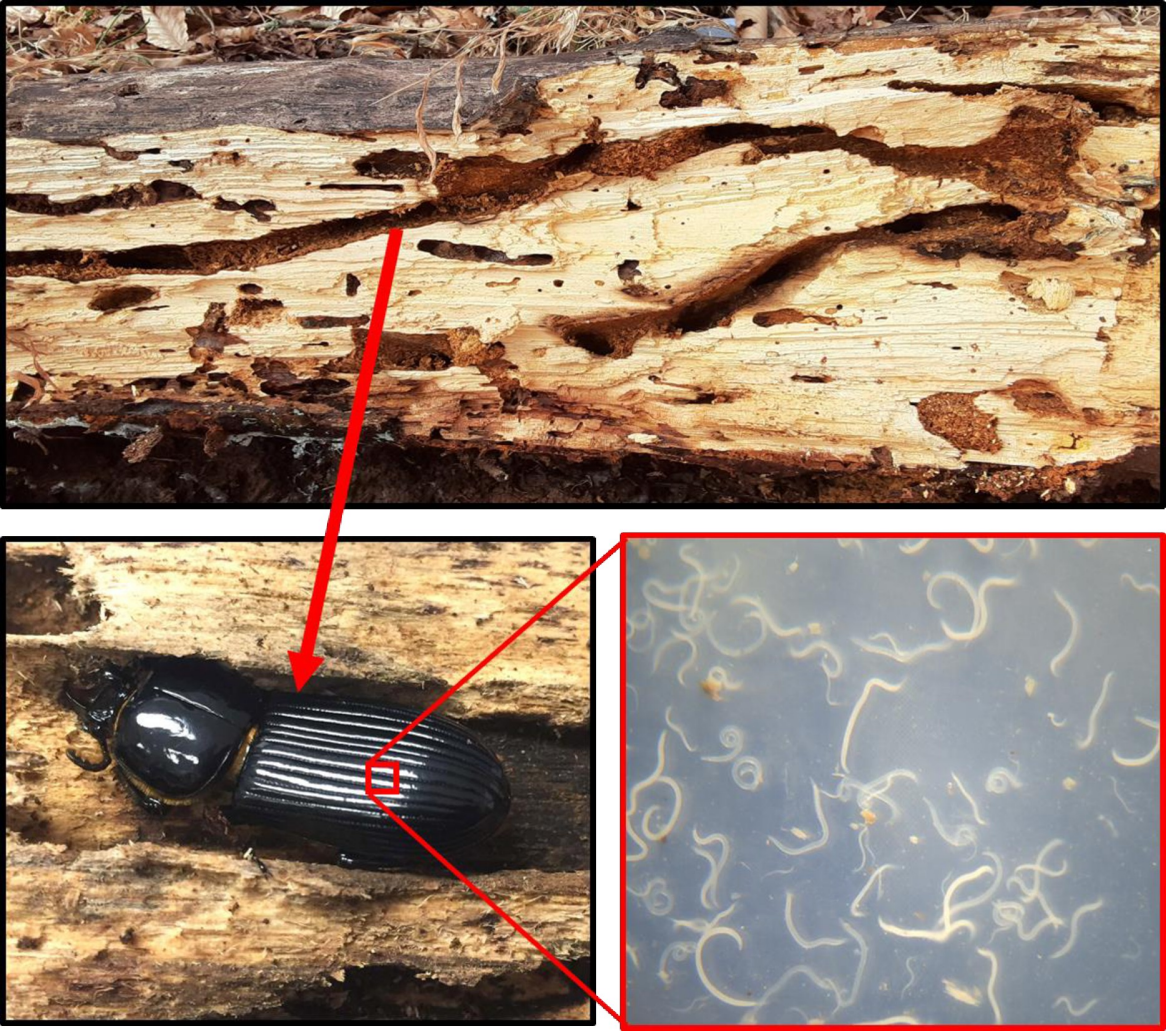
Horned passalus beetle, *Odontotaenius disjunctus*, in a burrow of a decaying hardwood log (top). Inset shows a collection of nematodes (*Chondronema passali*) taken from the hemocoel of the beetle and photographed in a petri dish. This parasite is common in all passalus beetle populations in the United States and individuals can have dozens to hundreds of nematodes. The mode of transmission of the nematode is unknown.

In the eastern United States, there is a common beetle that is host to an abundance of naturally-occurring parasites, making it well-suited to study how parasites affect anti-predator behavior; the horned passalus beetle, *Odontotaenius disjunctus* (Illiger, 1800; Fig 1), lives in decaying hardwood logs in forests throughout the eastern seaboard [27]. The beetles excavate tunnels in the logs where they raise offspring (grubs), and live their 1–2 year lives, consuming the wood, thereby aiding in the mechanical breakdown of the logs. This species is known to have a high degree of parental care, as the adults protect the grubs, provide macerated wood pulp for them, and even build cocoons for the pupae [28]. This species is host to a wide variety of external and internal parasites [29], including various mites, sometimes tachinid maggots, but importantly, they harbor an unusual nematode within their abdominal cavity (i.e. not the intestinal tract). *Chondronema passali* (Leidy, 1852; see Fig 1) is a species of nematode found only in this beetle, and it is extremely abundant, both at the population and individual level. It is found in high prevalence within every beetle population examined, and, beetles can have dozens to thousands of individual worms [30–33]. In the region of Georgia where we study this system, parasite prevalence is typically ~70% [34]. The worms are not associated with any one host organ and appear to live freely in the hemolymph, presumably pulling nutrients from the hemolymph to grow. Unfortunately, since it is not well-studied, the mode of transmission of this parasite is not yet known. However, it is believed that the beetles harbor only the larval stages of the nematode, and the adult worms live freely in the tunnel detritus [30,31], which presumably facilitates transmission of their progeny to other beetles.

Despite not knowing the full life cycle of *C*. *passali*, this host-parasite system has proven useful for study in our lab, simply because the sheer number of worms per beetle leads to many interesting questions about how hosts can live their lives being so burdened. Although this parasite is not lethal, it does have moderately negative impacts when the hosts need energy for a variety of events, including defending against attack [35], fighting conspecifics [36], wound-healing [37], mounting an immune reaction [38] or simply lifting heavy objects [39]. Importantly, one recent study also demonstrated that nematode-parasitized beetles appear to break down more wood during their lives [17], which could be construed as evidence that they are more motivated to eat, perhaps as a compensatory mechanism against the energy drain of the nematodes. Given this finding, an interesting follow-up question relates to the host behavior when dealing with perceived threats. If indeed the parasitized beetles are simply “hungrier”, does this mean they would be less motivated to adopt any anti-predator behavior that necessitates inactivity? Another follow-up question would be to evaluate if the impact of nematodes on female behavior differs from males; prior work with this system also indicated that female beetles are inherently more motivated to be active than males [34]. Here, we describe a series of experiments using this beetle-nematode system that were designed to address these questions, with results yielding a surprising answer.

## Methods

We report results from two identical experiments, which, importantly, were conducted in different phases of the horned passalus beetle life cycle. The first experiment was completed during summer (June-July) 2021, which overlaps with the beetle breeding and grub-rearing phase [32,33]. Here we tested a total of 140 beetles. Our second experiment was conducted during Jan-March 2022, which corresponds with the beetle wintering phase; by this time, all young from the prior summer and fall have completed metamorphosis, and adult beetles are mostly dormant in logs (author, *pers*. *obs*.). At this stage they are not in any form of physiological diapause, and when brought to the lab and placed in room temperature, they appear to resume normal activity and feeding (*pers*. *obs*.). In this experiment we tested 98 total beetles. All collection and laboratory procedures were the same in each experiment.

### Beetle collection and housing

For each experiment, beetles were hand-collected from nearby forested locations around Athens, GA, USA. We identified decaying hardwood logs and extracted adult beetles using hand tools. They were returned to the lab the same day and separated into individual plastic containers containing bits of decaying hardwood, which served as both refugia and a food source. After housing, all beetle containers were undisturbed for 1 week, to allow time to acclimate.

### Stressor treatments

**Fig 2:**
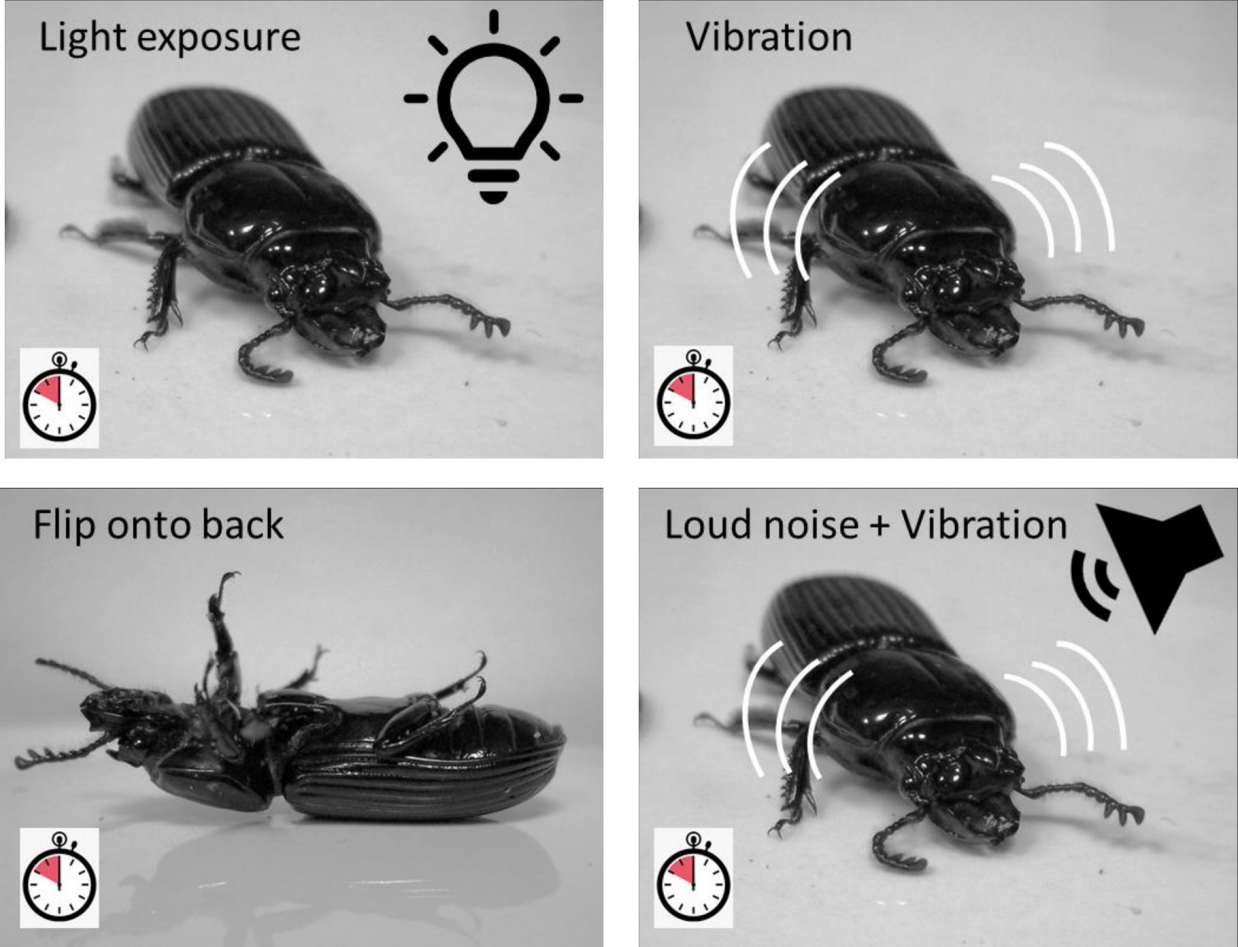
Stressor treatments applied to beetles to induce freezing behavior. Beetles were placed in a tabletop arena and exposed to one of four mild stressors, after which we timed the duration of freezing. Stressors included light exposure, flipping the beetle on its back, vibrating the arena (using a cell phone app) and vibration plus rapping the arena with a metal probe (loud sound). Each beetle was subjected to all four treatments on differing days (one treatment per day), and we averaged the durations of all treatments.

Each beetle was exposed to four different mild stressors over a week span, with one stressor applied each day. The different stressor types were applied in a predefined random order, so that the order of the treatments was not the same across all beetles. Testing was done in a lab room with the lights off for three treatments, but lights were on for one (below). On the day of testing, a beetle was removed from its container and placed in a shallow metal sorting tray (40cm x 25cm), which served as a testing arena. An observer applied the treatment of interest and then watched the beetles and recorded their behavior immediately thereafter (below). Following the treatments and observations, beetles were replaced back into their container until the next day of testing, and so on, until each had been exposed to the four treatments. In no particular order, the treatments were light exposure, flip upside down, vibration, and noise + vibration (Fig 2). Exposure to light was conducted when the room lights were on, and this involved merely removing the beetle from its refugia and placing it in the metal tray. We reasoned that this immediate exposure to light (for a beetle that spends its life in logs) would be an adverse stimulus, and elicit freezing behavior. All other treatments were conducted with the room lights off. The upside-down treatment involved placing the beetle on its back in the arena; beetles cannot self-right on a flat surface. The vibration treatment involved placing a cell phone under the metal tray, and using a cell phone app to vibrate the phone and tray. The beetles in the tray thus experienced vibration stimulus, which is known to trigger immobility or startle responses in other beetle species [40–42]. The last treatment was the vibration stimulus, plus where the observer sharply rapped 5 times on the side of the metal tray with a metal rod, which we reasoned would serve as an additional vibration and noise stimulus; in preliminary trials we noted that freezing can be induced with just the metal rod (see S1 Video).

### Assessing freezing behavior

An observer watched the beetles after each treatment for indications of freezing behavior. From preliminary trials we determined that these beetles react to such threats with an immediate cessation of forward movement, and where the beetle remains in place for an extended period of time (about 20 seconds on average, see results). The head may slowly move from side to side, and there can be slow antennal movement, but no overall body movement. There is no indication that they curl their legs and “huddle”, or even stiffen their appendages, which makes us believe this behavior is akin to a “freezing” response, whereby the animals simply stop moving to avoid detection [8]. According to the definition put forth by Sakai [43], this is consistent with a “fear response.” It may be that their lifestyle of living in burrows precludes the need to have a “thanatosis” or death-feigning behavior, or that their body morphology is not equipped for this. In fact, we have never witnessed any form of death-feigning in this beetle, after 10 years of study (Davis, *pers*. *obs*.). Nevertheless, the freezing behavior was easily observable with training; the observers for both experiments were trained to watch for this behavior and record (with a stopwatch) the start time, and end time, which was when the beetle began moving again (i.e. moving its body away from the initial position in the arena). This duration of freezing was the response variable of interest for our experiments, and in the end we had four recordings for each beetle. Importantly, some beetles showed no freeze response in some trials, and in these cases, we assigned them a zero for the duration. After all beetles had undergone the four different stressor treatments, we computed the average duration for each to use in analyses.

### Parasite assessment

After the beetles had been tested they were weighed with an electronic balance, and then euthanized with ethanol and dissected to determine levels of parasitism and gender. We carefully removed the abdominal plastron (under stereo magnification) and looked for worms of *C*. *passali*, which inhabit the abdominal hemocoel (Fig 1). Beetles can have dozens to hundreds of worms [33,44], and so we used a visual scoring system to simplify nematode assessment. Nematode burden was recorded on a 0–3 scale, where 0 = no worms present, 1 = fewer than 10 worms present, 2 = between 10 and 100, and 3 = more than 100 worms [34,36]. This same categorical scale has been used in multiple prior studies from our lab [35–37], and although crude, it does allow for a rapid visual assessment. At the same time, we identified the gender of the beetles based on the presence of the male aedeagus.

### Data analyses

Across both experiments we tested 238 beetles. Of these, the final tally of beetles assigned to each nematode group was n = 50, 67, 70 and 51. The duration of freezing variable was log-transformed to approximate a normal distribution. Beetle weight was normally-distributed, based on visual inspection of its histogram. To determine the possible predictors of freezing, we used a general linear model, with predictor variables including experiment number (1 or 2, included as a categorical factor), sex, parasite score (0, 1, 2, or 3, included as a categorical factor), and beetle weight was a continuous covariate. We also included a sex * parasite interaction term. Analyses were conducted using the Statistica 13.1 software package (Tibco Software, Inc.).

## Results

### General observations

While it was not the focus of the project, we first summarized the overall differences in the beetle reactions to the four different stressors. In the light exposure treatment, 35% entered a frozen state after the stimulus was applied. Meanwhile, 55% of beetles showed a freeze reaction after being flipped onto their back and 56% froze when exposed to substrate vibration. Finally, 59% responded (by freezing) to the combined vibration and noise treatment. The average duration of freezing across each treatment also reflects this variation, though these means include all beetles, even those that did not show any reaction (Table 1). In general, the longest freeze response was elicited by the flipping treatment, though the dual stressor treatment induced the most reactions.

**Table 1:** Summary of freeze times for different stressor treatments applied to passalus beetles in this study. See text of methods and Fig 2 for descriptions of each treatment. A total of 238 beetles were tested and each beetle underwent all four treatments (but on different days). The four immobility times were averaged for each beetle for statistical purposes. The grand mean shows the average of all 238 averages.

Stressor Treatment	N	Average time (sec)	Std.Dev.	Maximum
**Light exposure**	238	8	20.7	215
**Flip onto back**	236	31	74.6	671
**Vibration**	238	23	55.2	422
**Loud Noise + Vibration**	238	19	39.1	298
**Grand mean of means**	238	20	30.7	193

### Predictors of freezing

**Fig 3:**
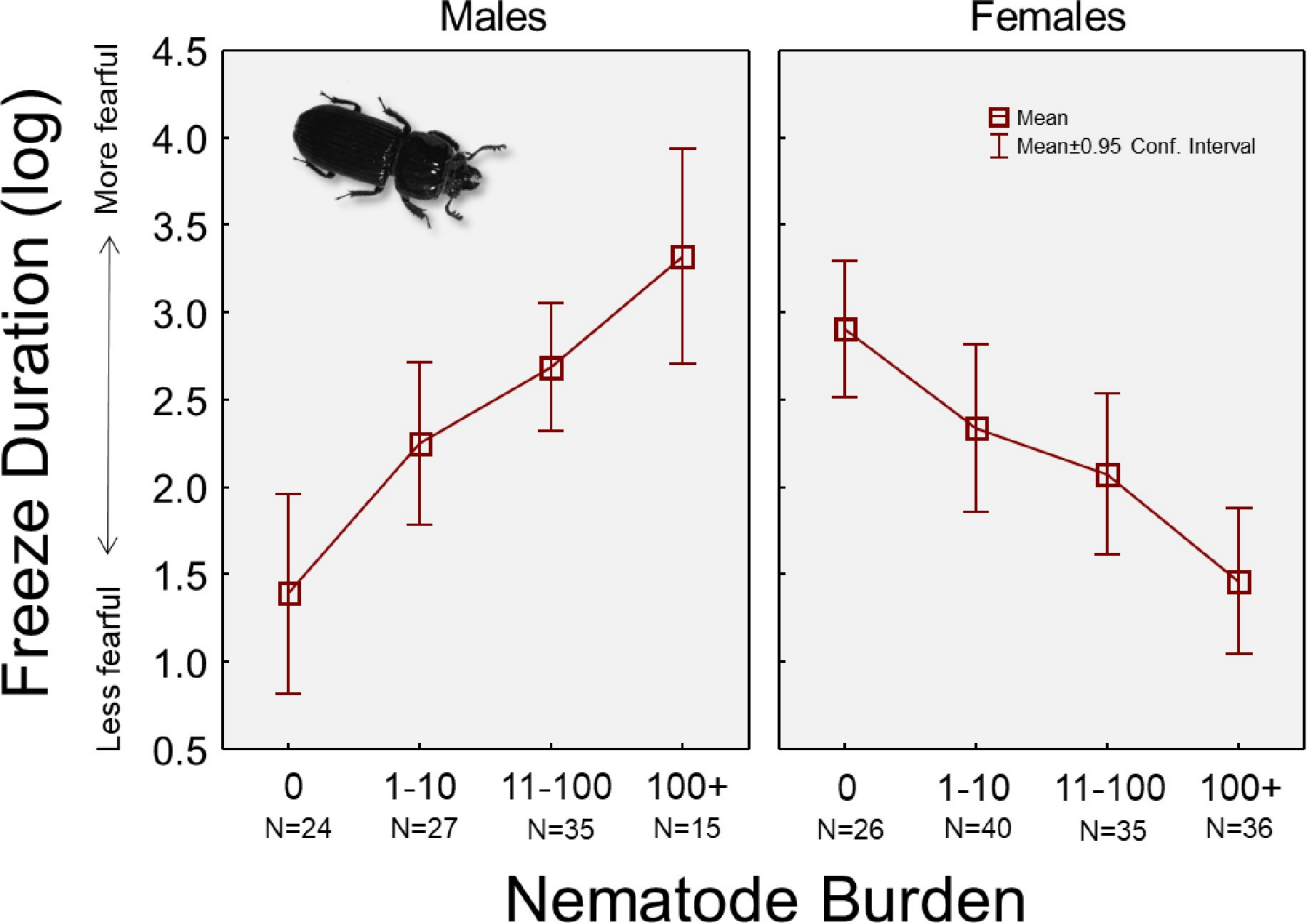
Mean freezing time for all beetles in each nematode infection category, and comparing males versus females. There was a significant sex*infection interaction term in the model examining predictors of immobility duration (see Table 2). Immobility (freezing) is generally associated with boldness/fearfulness, such that bolder or less fearful individuals tend to show short immobility times.

The GLM model that examined all predictors of freeze duration revealed no significant difference between the two experiments (p = 0.7566; Table 2), nor a main effect of beetle sex (p = 0.5449) or parasite load (p = 0.7937). Beetle mass was also not important for explaining variation in freeze behavior (p = 0.1950). Importantly, there was a significant sex*parasite interaction term (p<0.0001), which is illustrated in Fig 3. This graph shows a striking difference in how increasing burdens of nematodes affected male versus female passalus beetles; male beetles tended to increase the duration of freezing as parasite burdens increased, while females appeared to reduce their freezing behavior with increasing levels of parasitism.

**Table 2:** Summary of ANCOVA model examining all potential predictors of freeze time (in seconds, log-transformed) displayed by horned passalus beetles in response to mild stressors. Parasite load refers to the 0–3 scale used to assess host burden of *C*. *passali* nematodes. The significant parasite*sex interaction is displayed in Fig 3.

Predictor	SS	df	MS	F	p
**Experiment #**	0.15	1	0.15	0.10	0.7566
**Parasite Load**	1.60	3	0.53	0.34	0.7937
**Beetle Sex**	0.57	1	0.57	0.37	0.5449
**Beetle Weight**	2.62	1	2.62	1.69	0.1950
Parasite Load*Sex	72.20	3	24.07	15.49	<0.0001
**Error**	354.15	228	1.55		

## Discussion

Horned passalus beetles live with many different types of external and internal parasites [29], though the *C*. *passali* nematode is particularly noteworthy for the sheer number of worms that can inhabit individual beetles. Visually, there appears to be no immune response to the nematodes, since we have never observed melanized or killed worms during dissections (and see S2 Video). However, as nematodes living in the hemolymph, we can reason that the worms must be absorbing nutrients and resources from the blood, thereby limiting what the host can use. In prior experiments in our lab, we determined that this parasite exerts its strongest influence during times when hosts require brief energy bursts, such as during stress events [35,38,39], and wound healing [37], where it (the nematode) appears to moderately reduce host performance. Thus, we undertook this study to ascertain if the nematode similarly influences a behavior that seemingly requires no energy, since by definition, freezing is the cessation of movement (although we did not actually measure energy or metabolic activity during freezing). Further, we did not specifically anticipate that the impact would be positive or negative, since in theory, parasites could cause either scenario, either by causing lethargy (unwillingness to move) or by heightening motivation to forage (to compensate for energy drain) [16]. Regardless of the direction, we reasoned that any effect of this parasite would be consistent across all beetles, and so we were not expecting to discover these distinct differences between sexes in this behavior. In fact, based on our review of the relevant literature on all forms of anti-predator behaviors (thanatosis, tonic immobility, freezing, etc.), we believe this to be the first-described case of a parasite that influences this behavior, and, in different directions within male and female hosts.

We are confident that the findings of this project are not due to random chance for two reasons. First, the approach we used–whereby the beetle nematode burden was not known until after all behavior tests were complete–ensured a completely objective and unbiased outcome. Second, we found qualitatively similar results from two different experiments (see S1 File). Interestingly, the fact that similar results were found in our summer experiment, and our winter experiment, also demonstrates that this pattern is not related to the energy cost of rearing and caring for young.

In addition to the reasoning given above, we can also eliminate a wide variety of potential biological explanations, based on prior work in our lab and others where male and female passalus beetles were studied (Table 3). First, male and female beetles appear to be equally parasitized by *C*. *passali*, both in terms of prevalence and individual burden [34,44]. Most of the physiological and biological functions evaluated thus far also appear to show that males and females do not differ substantially, especially in terms of energy use; baseline metabolic rate [37] and heart rate [38,45] are both similar between the sexes. The overall feeding rate of males and females (indexed by how much wood is broken down) is also similar between males and females [17], consistent with their similar metabolism. There is no indication that the immune system of males and females differ either, based on hemocyte density [38]. Behaviorally, there are a few sex differences, such as a greater propensity in females to explore than do males [34], and females give more frequent alarm calls than males when attacked. Both of these could be indicators of greater overall boldness in females, though taken with the results from the current study, we now wonder if these prior findings were the result of the parasite, and not the beetle gender *per se*.

**Table 3:** Summary of all known male-female differences in behavior, physiology and parasitism in horned passalus beetles, *Odontotaenius disjunctus*.

Category	Variable/Behavior	Outcome	Source
Parasite Biology	Nematode prevalence	Females = males	[34]
Parasite Biology	Host nematode burden	Females = males	[44]
**Host Biology**	**Body size**	**Females>males**	**[32,44]**
**Host Biology**	**Walking/exploration behavior**	**Females>males**	**[34]**
Host Biology	Wood breakdown rate	Females = males	[17]
Host Biology	Physical lifting/pulling strength	Females = males	[39,44,46]
Host Biology	Fighting ability	Females = males	[36]*
**Stress behavior**	**Alarm call rate during simulated attack**	**Females>males**	**[35]**
Stress behavior	Intensity of struggle during simulated attack	Females = males	[35]
Host Physiology	Baseline metabolic rate	Females = males	[37]
**Host physiology**	**(mass-specific) metabolic rate during wound-healing**	**Females>males**	**[37]**
Host Physiology	Hemocyte density	Females = males	[38]
Host Physiology	Heart rate	Females = males	[45]
Host Physiology	Heart rate elevation during stress	Females = males	[45]
Host Physiology	Water content	Females = males	[28]

*Male-female differences were not reported in the paper but the raw data did indicate no difference.

Regarding the behavioral differences between sexes, perhaps the most important indicator to focus on is the freeze reactions of beetles that were *not* parasitized by *C*. *passali* nematodes. It appears that male and female horned passalus beetles may have innately differing styles of dealing with stressors, as noted from the groups with no nematodes (Fig 3). Of these beetles, females appear to remain in a longer freeze state than males (female average = 34 sec, male average = 14 sec), indicating that in that absence of nematodes, females are *inherently* more fearful, while unparasitized males tend to be more bold. By extension, this could mean that each sex adopts differing means of dealing with predators when parasitized, though the exact mechanism is not clear. Interestingly, the idea that sexes can differ in their reaction to predator stressors is not new; Lagos and Herberstein [47] demonstrated how male crickets have larger elevations in metabolism than females do when presented with predator cues. Similarly, male fruitflies had stronger metabolic responses to a stressor than did females [48]. However, since the opposite pattern was found in our study (female beetles were more fearful), this phenomenon may be species-specific.

Clearly, this project raises many questions that deserve additional investigation. For example, future efforts could investigate whether the worms alter concentrations of any biological compounds within the hemolymph that could affect host behavior, such as amines, which are involved in fight or flight behaviors in insects [49–51]. Increases or decreases in concentrations of amines such as octopamine, dopamine or serotonin can affect behaviors similar to what we studied here. Given that the magnitude of changes in freezing behaviors we observed generally matched the intensity of nematode burdens (but differently for males and females), this implies a dose, or concentration effect. That is, more (or less) parasitic nematodes leads to greater host behavioral changes. Therefore, examining amine concentrations of male and female beetles with varying nematode burdens should provide insights.

Despite not yet knowing exactly how the parasite effect arises, we can at least draw inferences or generalities about the ultimate behavior of each beetle sex when heavily parasitized. Given that longer freezing or tonic immobility is generally associated with greater fearfulness [52,53], heavily parasitized male beetles would likely not be motivated to explore or risk exposure to predators by leaving their burrow–either to enter new burrows in their log, or to leave the log entirely in search of a new one. Conversely, our results show heavily-parasitized females appear to be more bold, and intuitively, this means they would be more willing to risk venturing outside their home burrow, or even their log. In fact, this reasoning is consistent with the prior work in our lab, where female beetles were found to be 30% more willing to explore a novel environment than males were [34], and consider also that 70% of horned passalus beetles in nature are parasitized with *C*. *passali*.

Given the conclusion above, we believe is possible that this is an example of parasite manipulation of host behavior, designed to improve its transmission [54], which other nematodes are known to do [55]. However, as pointed out by Lafferty and Shaw [16], finding concrete evidence for direct host manipulation by parasites is often elusive. The transmission of *C*. *passali* has been suggested (but without evidence) to involve mature larval worms exiting the hosts during the host egg-laying period, which is in the early summer [30]. If this is true, then it is possible that the nematode could be specifically manipulating female beetles to become less risk-averse so they would seek out new burrows or logs to lays eggs in, thereby enhancing its own transmission. However, it is important to point out that *C*. *passali* inhabits a region of the body removed from any neural activity (the hemocoel), as opposed to being localized in the brain region where it may be easier to alter neural activity [16]. Though in other nematodes, host manipulation is facilitated by altering serotonin signaling in the host brain [55]. In the end, determining whether this case represents definitive “host manipulation” or an artifact of some inherent host sickness behavior is challenging, and this is problematic in other nematode-host systems too [56]. Moreover, if this were true (*C*. *passali* is affecting host behavior), it would also mean that the nematode manipulates male beetles to be more risk-averse and sedentary, for unknown reasons.

To directly test if the pattern we discovered represents actual manipulation of host beetles by *C*. *passali*, would require the ability to experimentally infect naïve hosts with the nematode, which we cannot yet do, because of the many unanswered questions around its transmission. Moreover, a confounding issue with using naturally-occurring nematode infections in wild hosts, is that we cannot know for sure the age of the beetles. It is very possible that nematodes build up in individual beetles as they age, and, if age affects host behavior, this could confound interpretation of the “parasite effects” on behavior. This has been shown with species of crickets [57], where males became less bold as they aged, but females did not. The authors of that paper thought this was due to the predation risk associated with calling for mates (which is not the same in this system). From a logistical standpoint, conducting such an investigation on age effects would be difficult in our beetle system. This would require having known-aged beetles to test, which could only be possible by rearing them in captivity, and this alone is difficult with this species, since young grubs are raised by their parents [33]. Second, and again, we do not yet know how to experimentally infect hosts with this nematode.

More generally, these results highlight an important knowledge gap in the body of research around anti-predator behaviors, which is the role of parasites, and how they have the potential to influence the host reactions to predators or related threats. To our knowledge, no prior studies of this topic have considered this potential factor, either in the study design, or when interpreting results. Indeed, in a thorough review of the topic (of 91 studies), no mention was made of this [8]. Further, the knowledge that parasites can even alter behaviors of males and females differently, could also have implications for other projects where anti-predator behaviors have been compared between sexes [58,59]. Given that ours is the first study to bring this issue to light, this is clearly a nascent topic needing of more exploration.

## Conclusions

We exposed wild-caught horned passalus beetles to four different mild stressors and recorded how long they freeze (cease movement) in response. After pairing these data with the beetle nematode burdens, we discovered that heavily parasitized female beetles reacted much differently than heavily parasitized males to the same stressors. Female beetles with heavy nematode burdens appear to be more bold than similarly-parasitized males, which itself engenders questions about how this affects their annual cycle. There are few known physiological differences in the beetle sexes that could explain the sex difference, and therefore more investigation is needed to fully elucidate the mechanism. This discovery highlights the importance of understanding the impact of parasites to anti-predator behaviors in animals, a topic which has been neglected.

## Supporting information

S1 Video Video segment showing a horned passalus beetle freezing briefly after an observer gently rapped on the tray. (MP4)

S2 Video (MP4)

S1 File Document containing figures that display results from each experiment separately. (DOCX)

S1 Raw data Excel file containing complete dataset generated from this study. (XLSX)
