# Supplementary material for: Sex-specific effects of a parasite on stress-induced freezing behavior in a natural beetle-nematode system
Source: PLoS One. 2023 Mar 14;18(3):e0281149. doi: 10.1371/journal.pone.0281149 (PMC10013888; doi:10.1371/journal.pone.0281149)

Supplemental file for **Sex-specific effects of a parasite on anti-predator freezing behavior in a natural beetle-nematode system**

Figure S1. Comparison of findings from two identical experiments, conducted during summer 2021 (beetle breeding season) and winter 2022 (non-breeding season).


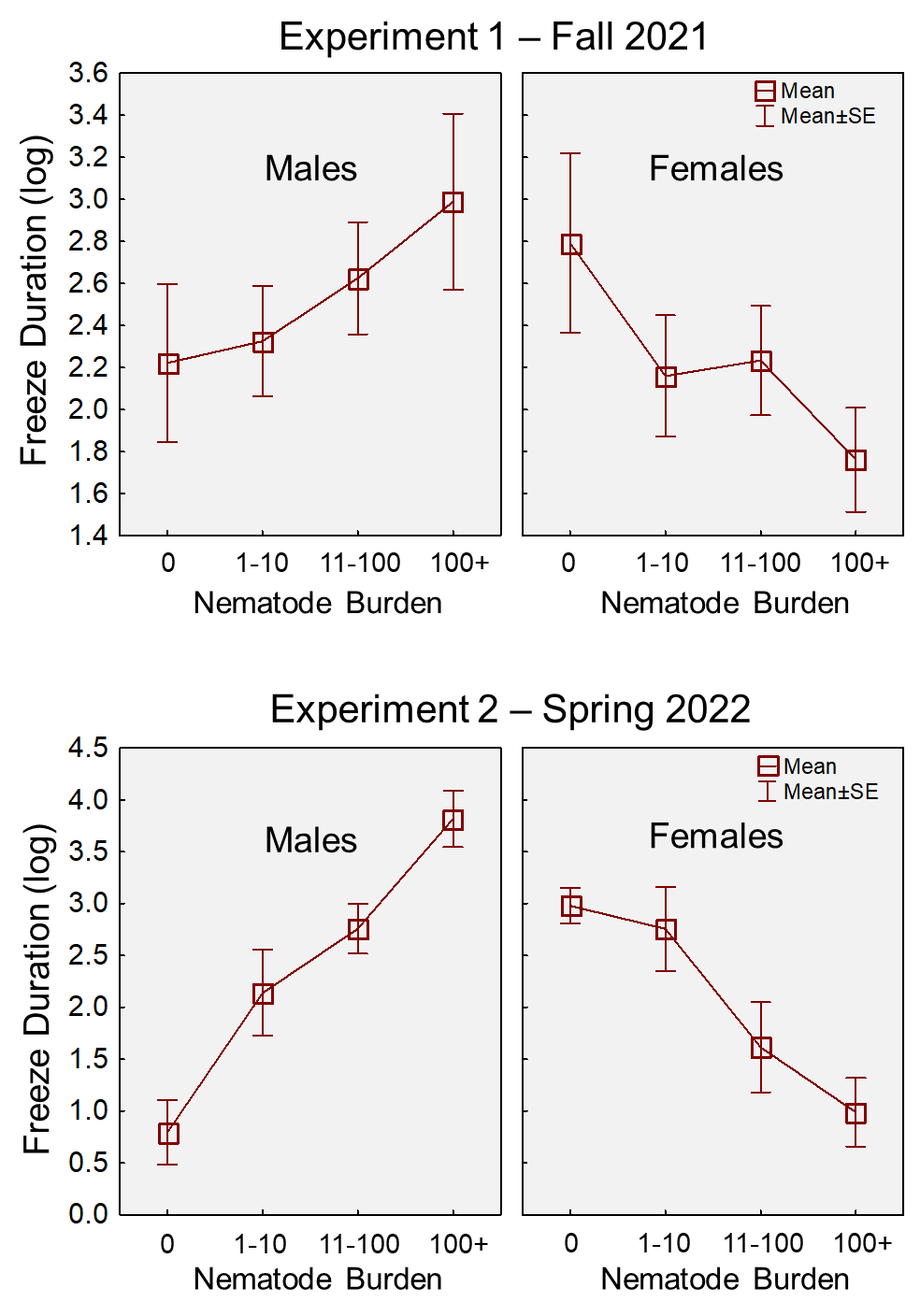

Supplement: S1 File — (DOCX) [file pone.0281149.s003.docx]
